# Cannabidiol/cannabigerol exposure and neuropathy-related outcomes after neurotoxic chemotherapy: a real-world cohort study

**DOI:** 10.3389/fpain.2026.1945720

**Published:** 2026-09-22

**Authors:** Nicholas M. Graziane, Ajay Macherla, Matthew Giampetro

**Affiliations:** 1 Departments of Anesthesiology and Perioperative Medicine and Neuroscience and Experimental Therapeutics, Penn State College of Medicine, Hershey, PA, United States; 2 Department of Anesthesiology and Perioperative Medicine, Penn State College of Medicine, Hershey, PA, United States

**Keywords:** cannabidiol, cannabigerol, cannabinoids, chemotherapy-induced peripheral neuropathy, real-world evidence, TriNetX

## Abstract

**Background:**

Chemotherapy-induced peripheral neuropathy (CIPN) is a common complication of neurotoxic chemotherapy, yet effective pharmacologic options for both prevention and treatment of established painful CIPN remain limited. Non-delta-9-tetrahydrocannabinol (THC) cannabinoids and related minor cannabinoids show antinociceptive effects in preclinical CIPN models, but their real-world clinical relevance remains unclear.

**Methods:**

We performed a retrospective secondary analysis of de-identified electronic health record (EHR) data using the TriNetX Research Network to examine whether documented cannabidiol/cannabigerol (CBD/CBG) exposure was associated with subsequent neuropathy-related diagnoses among adults receiving neurotoxic chemotherapy. Patients with neuropathy-related diagnoses on or before chemotherapy were excluded. Two Cox proportional hazards analyses were performed. The post-chemotherapy exposure analysis compared post-chemotherapy CBD/CBG exposure with post-chemotherapy exposure to gabapentin, pregabalin, or duloxetine. The pre-chemotherapy exposure analysis compared patients with documented CBD/CBG exposure within one year before chemotherapy to patients who had no documented CBD/CBG exposure during that pre-chemotherapy window. The outcome was a composite neuropathy-related diagnosis 30–365 days after the index event.

**Results:**

In the adjusted post-chemotherapy exposure model, 83 CBD/CBG-exposed patients were compared with 118,001 active-comparator patients. Post-chemotherapy CBD/CBG exposure was associated with lower hazard of subsequent neuropathy-related diagnosis-code outcomes (Hazard ratio [HR] = 0.162, 95% confidence interval [CI] = 0.041–0.649, *p* = 0.010). In the adjusted pre-chemotherapy exposure model, 162 CBD/CBG-exposed patients were compared with 633,329 controls. Pre-chemotherapy CBD/CBG exposure was not significantly associated with neuropathy-related diagnosis-code outcomes (HR = 0.859, 95% CI = 0.476–1.551, *p* = 0.614).

**Conclusions:**

Post-chemotherapy CBD/CBG exposure was associated with lower observed hazard of neuropathy-related diagnosis-code outcomes, whereas pre-chemotherapy exposure was not significant. These exploratory findings require cautious interpretation because of sparse exposed-arm events, EHR exposure limitations, and potential competing-risk bias.

## Introduction

Chemotherapy-induced peripheral neuropathy (CIPN) is a common and clinically significant adverse effect of several widely used anticancer agents, including platinum compounds, taxanes, vinca alkaloids, and proteasome inhibitors (1–3). The prevalence of CIPN varies depending on the type of chemotherapy, with platinum derivatives such as cisplatin and oxaliplatin showing the highest rates, ranging from 70% to 100% (4). CIPN commonly presents with numbness, tingling, paresthesia, dysesthesia, mechanical hypersensitivity, and neuropathic pain, which may persist long after chemotherapy has ended (1, 5). In addition to reducing quality of life, CIPN can interfere with cancer care by necessitating chemotherapy dose reduction or discontinuation, thereby creating a difficult balance between optimal cancer treatment and long-term neurologic toxicity (1). Because cancer survivorship continues to improve, identifying effective strategies to prevent or treat CIPN remains an important unmet clinical need.

Despite the clinical burden of CIPN, available pharmacological options remain limited. No medication is currently recommended as an established preventive therapy, and treatment options for established painful CIPN are modest (6). The American Society of Clinical Oncology guideline identifies duloxetine as the only agent with appropriate evidence supporting its use for established painful CIPN, while also noting that the magnitude of benefit is limited (6). Other medications commonly used for neuropathic pain, including gabapentin and pregabalin, are frequently used empirically in clinical practice but have not shown sufficient evidence to serve as established CIPN-directed therapies (1, 6).

Cannabinoid-based therapies have been used clinically or evaluated for several pain-related conditions, including chronic and neuropathic pain, cancer pain, and multiple sclerosis-associated symptoms, but their analgesic effects are generally modest and evidence for CIPN remains limited and inconclusive (7–9). In addition, delta-9-tetrahydrocannabinol (THC)-containing or THC-dominant approaches remain limited by the psychoactive and euphorigenic effects of THC, as well as acute cognitive and psychomotor effects involving attention, memory, perception, sedation, dizziness, and motor performance (10). Clinical translation is further complicated by product heterogeneity, regulatory barriers, and limited CIPN-specific efficacy data. For example, a small randomized crossover pilot trial of nabiximols, a 1:1 THC:cannabidiol (CBD) oromucosal extract, did not significantly reduce CIPN compared with placebo despite responder signals in a subset of participants (11–14). Notably, the cannabis plant produces numerous phytocannabinoids beyond THC (15). These include non-euphorigenic or minimally euphorigenic compounds often referred to as minor cannabinoids, which in rodent models of CIPN have shown antinociceptive efficacy (16–24). This supports the possibility that non-THC cannabinoids may engage pain-modulatory mechanisms relevant to chemotherapy-associated neuropathy.

Real-world electronic health record datasets provide an opportunity to identify clinical signals that may inform preclinical and translational research priorities. Such analyses cannot establish causality and are limited by coding accuracy, medication capture, confounding by indication, and incomplete information about dose, formulation, and adherence. However, when used carefully, real-world datasets can help determine whether a therapeutic class shows enough clinical signal to justify mechanistic testing under controlled experimental conditions. In the present study, we used the TriNetX Research Network to examine whether documented CBD/cannabigerol (CBG)-related EHR exposure was associated with reduced neuropathy-related diagnoses among adults receiving neurotoxic chemotherapy. We evaluated two complementary questions: first, whether CBD/CBG-related exposure after chemotherapy was associated with reduced subsequent neuropathy-related diagnoses compared with traditionally used neuropathic-pain medications; and second, whether cannabinoid exposure before chemotherapy was associated with reduced later neuropathy-related diagnoses. This approach was designed to determine whether real-world clinical data support continued preclinical investigation of minor, non-THC, cannabinoids as candidate therapeutics for CIPN.

## Methods

### Data source

This retrospective cohort study was conducted using the TriNetX Research Network, a federated electronic health record database that provides access to de-identified patient-level clinical data from participating healthcare organizations. TriNetX includes structured data on demographics, diagnoses, procedures, medications, laboratory values, and other clinical encounters. At the time of analysis, the Research Network included 115 healthcare organizations (HCOs). The updated Cox proportional hazards analyses were generated in TriNetX on August 24, 2026. No calendar-year restriction was applied. The analyses used all available structured EHR data in the TriNetX Research Network that met the cohort definitions at the time the queries were performed. All analyses were performed within the TriNetX analytics platform using de-identified data. Because the data were de-identified and no direct patient interaction occurred, this study did not constitute human subjects research requiring informed consent.

### Study design

Two complementary retrospective cohort analyses were performed to evaluate whether documented CBD/CBG-related EHR exposure was associated with reduced neuropathy-related diagnoses after neurotoxic chemotherapy. The first analysis evaluated a post-chemotherapy exposure design, comparing patients with CBD/CBG exposure after chemotherapy with patients treated with commonly used neuropathic-pain medications after chemotherapy (Figure 1A). The second analysis evaluated a pre-chemotherapy exposure design, comparing patients with CBD/CBG exposure before chemotherapy with patients without documented pre-chemotherapy CBD/CBG exposure (Figure 1B). Both analyses used Cox proportional hazards models to estimate adjusted hazard ratios for subsequent neuropathy-related diagnoses.

**Figure 1:**
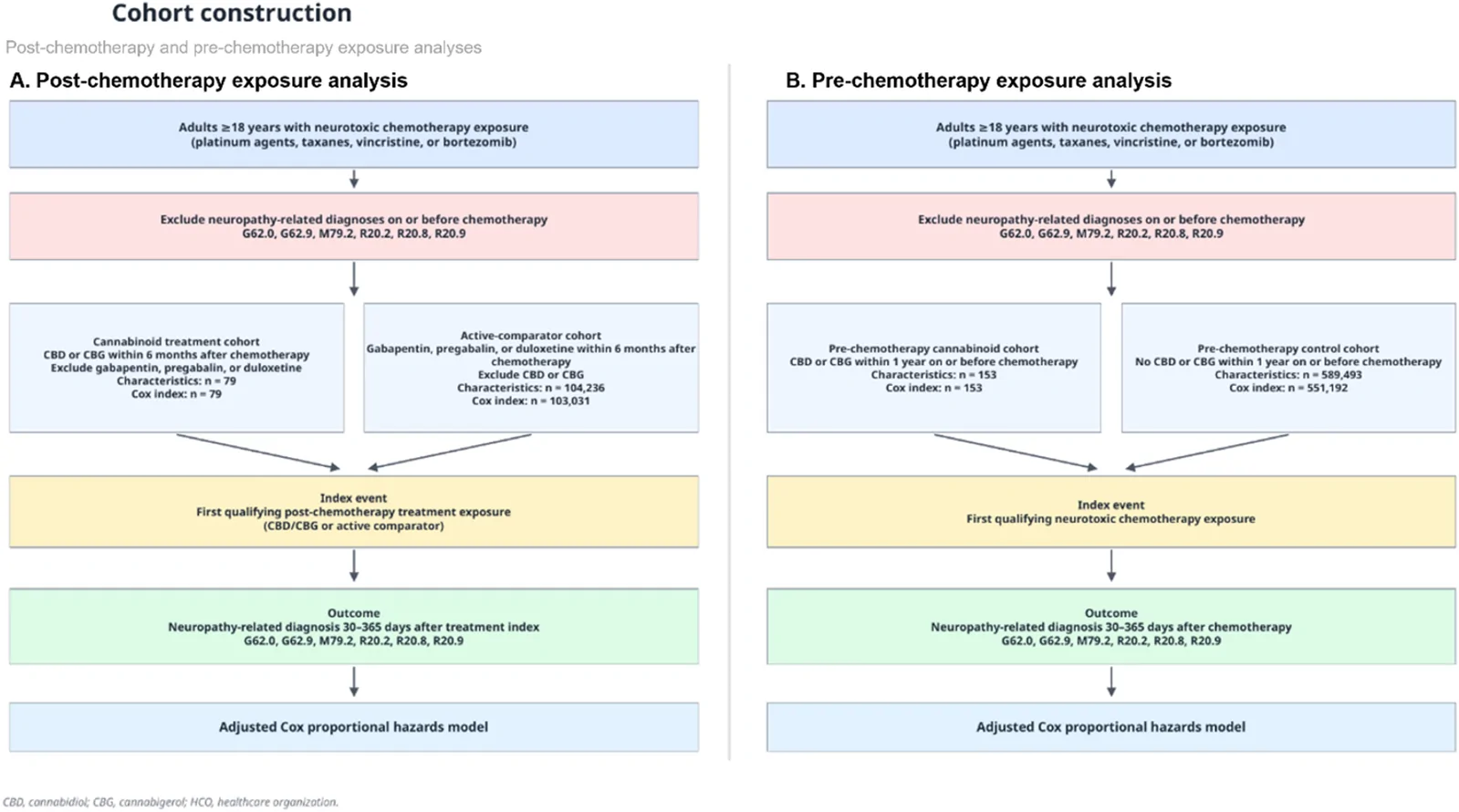
Cohort construction for post-chemotherapy exposure and pre-chemotherapy exposure analyses. Adults receiving neurotoxic chemotherapy were identified in the TriNetX Research Network. Patients with neuropathy-related diagnoses on or before chemotherapy were excluded. **(A)** In the post-chemotherapy exposure analysis, patients with CBD/CBG exposure within 6 months after chemotherapy were compared with patients receiving gabapentin, pregabalin, or duloxetine within the same post-chemotherapy window. **(B)** In the pre-chemotherapy exposure analysis, patients with CBD/CBG exposure within 1 year before chemotherapy were compared with patients without documented CBD/CBG exposure in that window. Neuropathy-related outcomes were assessed 30–365 days after the index event using adjusted Cox proportional hazards models.

CBD and CBG were analyzed together because both are non-THC cannabinoids with preclinical relevance to chemotherapy-induced neuropathy and because structured EHR capture of individual CBD-only and CBG-only exposure was too sparse to support stable separate primary models. Therefore, the exposure should be interpreted as documented CBD/CBG-related EHR exposure rather than exposure to a single commercial product, dose, or formulation. Structured EHR medication concepts do not reliably capture whether the documented exposure reflected isolated CBD, isolated CBG, a CBD-rich or CBG-containing product, or a broader cannabinoid formulation. Accordingly, throughout the manuscript, CBD/CBG exposure refers to structured EHR documentation of CBD or CBG concepts and should not be interpreted as confirmation that patients used isolated CBD-only or CBG-only products.

### Study population

Eligible patients were adults aged 18 years or older with documented exposure to at least one neurotoxic chemotherapy agent. Neurotoxic chemotherapy exposure was defined using medication and procedure concepts for platinum compounds, taxanes, vinca alkaloids, and proteasome inhibitors. The final chemotherapy concept set included structured medication and procedure concepts for platinum compounds, taxanes, vinca alkaloids, and proteasome inhibitors, specifically cisplatin, oxaliplatin, carboplatin, paclitaxel, protein-bound paclitaxel, docetaxel, vincristine, and bortezomib. Medication and chemotherapy administration concepts were identified using RxNorm, a normalized clinical drug terminology for medications, and Healthcare Common Procedure Coding System (HCPCS) terms available in TriNetX, as listed in Supplementary Table A. These terms were used consistently across both analyses.

To reduce capture of pre-existing neuropathy, patients were excluded if they had a neuropathy-related diagnosis on or before the qualifying chemotherapy exposure. Baseline neuropathy exclusion terms included drug-induced polyneuropathy, paresthesia of skin, other disturbances of skin sensation, unspecified disturbances of skin sensation, polyneuropathy unspecified, and neuralgia/neuritis unspecified. Codes used for exclusion and outcome definitions are listed in Supplementary Table B.

### Post-chemotherapy cannabinoid analysis

For the post-chemotherapy exposure analysis, patients were required to have neurotoxic chemotherapy and no neuropathy-related diagnosis on or before chemotherapy. The CBD/CBG-exposed cohort included patients with documented CBD or CBG exposure within 6 months on or after neurotoxic chemotherapy. To create a mutually exclusive cannabinoid cohort, patients in this group were excluded if they had documented gabapentin, pregabalin, or duloxetine exposure in the same post-chemotherapy treatment window. Exposure and comparator concepts are listed in Supplementary Tables C and D.

The active-comparator cohort included patients with documented gabapentin, pregabalin, or duloxetine exposure within 6 months on or after neurotoxic chemotherapy. Patients in the active-comparator cohort were excluded if they had documented CBD or CBG exposure. The purpose of this design was to compare post-chemotherapy cannabinoid exposure with commonly used neuropathic-pain medications rather than with an untreated control group, consistent with active-comparator approaches used to reduce confounding by indication in observational pharmacoepidemiology (25–27). The descriptive characteristics export included 83 patients in the CBD/CBG cohort and 120,807 patients in the active-comparator cohort. After applying Cox model index-date and time-at-risk requirements, 83 and 118,001 patients, respectively, were included in the Cox proportional hazards analysis.

For the Cox proportional hazards model, both cohorts were indexed at initiation of the first qualifying post-chemotherapy treatment. In the cannabinoid cohort, this was the first documented CBD/CBG exposure after neurotoxic chemotherapy. In the active-comparator cohort, this was the first documented gabapentin, pregabalin, or duloxetine exposure after neurotoxic chemotherapy. Outcomes were assessed 30 to 365 days after the cohort-specific treatment index date.

### Pre-chemotherapy cannabinoid exposure analysis

For the pre-chemotherapy exposure analysis, patients were required to have neurotoxic chemotherapy and no neuropathy-related diagnosis on or before chemotherapy. The exposed cohort included patients with documented CBD or CBG exposure within 1 year on or before neurotoxic chemotherapy. The control cohort included patients with neurotoxic chemotherapy and no documented CBD or CBG exposure within 1 year on or before chemotherapy. Gabapentin, pregabalin, and duloxetine were not exclusion criteria in this analysis but were handled as covariates as described below. The pre-chemotherapy analysis included 162 patients in the CBD/CBG cohort and 662,672 patients in the control cohort at query definition, with 633,329 control patients entering the Cox model after index-date and time-at-risk requirements.

For the pre-chemotherapy analysis, the index event was defined as the first qualifying neurotoxic chemotherapy exposure. Outcomes were assessed 30 to 365 days after chemotherapy.

### Outcome definition

The primary outcome was a composite neuropathy-related diagnosis occurring after the index event. The same outcome definition was used for both analyses. The composite included International Classification of Diseases, Tenth Revision, Clinical Modification (ICD-10-CM) diagnosis codes for drug-induced polyneuropathy, polyneuropathy unspecified, neuralgia and neuritis unspecified, paresthesia of skin, other disturbances of skin sensation, and unspecified disturbances of skin sensation. These corresponded to G62.0, G62.9, M79.2, R20.2, R20.8, and R20.9 (Supplementary Table B). The outcome window was defined as 30 to 365 days after the index event. A 30-day lag was used to reduce capture of diagnoses recorded at the same visit or during the immediate peri-index period and to better identify neuropathy-related diagnoses emerging after chemotherapy or after treatment exposure.

### Covariates

Covariates were selected based on clinical relevance to neuropathy risk, treatment selection, and cannabinoid prescribing. Covariates were measured before the index event, ending 1 day before the index date. The covariate window did not include events occurring on the same day as the index event to reduce capture of diagnoses or medications documented as part of the index encounter.

Both Cox models included age at index, sex, diabetes mellitus, seizure-related diagnoses, metastatic cancer, and opioid analgesic exposure. Diabetes was included because it is an established risk factor for peripheral neuropathy and could influence subsequent neuropathy diagnosis. Seizure-related diagnoses were included because prescription CBD is commonly used in seizure disorders, creating potential confounding by indication. Metastatic cancer diagnoses were included as markers of cancer burden and disease severity. Opioid analgesic exposure was included as a marker of baseline pain burden and symptom complexity. Covariate concepts and associated coding are listed in Supplementary Table E.

For the pre-chemotherapy analysis, gabapentin, pregabalin, and duloxetine were additionally included as covariates because pre-index use of these medications may indicate baseline pain, neuropathic symptoms, mood disorders, or other comorbidities that could influence subsequent neuropathy-related diagnosis. For the post-chemotherapy active-comparator analysis, gabapentin, pregabalin, and duloxetine were not included as covariates in the final model because they were used to define the comparator cohort and were therefore exposure-defining rather than independent adjustment variables.

### Statistical analysis

Cox proportional hazards regression models were used to estimate adjusted hazard ratios and 95% confidence intervals for neuropathy-related diagnoses. Cohort membership was included as the primary independent variable. Hazard ratios less than 1 indicated lower hazard of neuropathy-related diagnosis in the CBD/CBG cohort relative to the comparator cohort, whereas hazard ratios greater than 1 indicated higher hazard. Models were run within the TriNetX analytics environment using de-identified, aggregated real-world data. Statistical significance was set at *p* < 0.05.

Because the CBD/CBG-exposed cohorts were substantially smaller than the comparator cohorts, estimates were interpreted with attention to confidence interval width and cohort size. Furthermore, small exposure cohorts may generate less precise estimates. Therefore, analyses were interpreted as exploratory and hypothesis-generating rather than causal.

Descriptive outcomes queries were also performed to summarize the number and proportion of patients with neuropathy-related diagnosis-code outcomes in each cohort using the same composite outcome definition and 30- to 365-day post-index window used for the Cox proportional hazards models. These descriptive event counts were used to contextualize model precision and sparse-event concerns.

As a descriptive *post hoc* competing-risk check requested during review, death was assessed within 0 to 365 days after the post-chemotherapy exposure index in the CBD/CBG and active-comparator cohorts. This analysis was descriptive only and was not modeled as a formal competing-risk regression. Therefore, mortality findings were used to contextualize interpretation of neuropathy-related diagnosis-code outcomes rather than to generate adjusted competing-risk estimates.

This study was reported in accordance with the STROBE guideline for observational studies. The completed checklist is provided as Additional file 1.

## Results

### Cohort identification and participant characteristics

The post-chemotherapy exposure analysis compared post-chemotherapy CBD/CBG exposure with active-comparator treatment using gabapentin, pregabalin, or duloxetine. The pre-chemotherapy exposure analysis compared patients with documented CBD/CBG exposure within one year before chemotherapy to patients who had no documented CBD/CBG exposure during that pre-chemotherapy window.

In the post-chemotherapy exposure analysis, the CBD/CBG characteristics cohort included 83 patients from 24 HCOs, while the active-comparator characteristics cohort included 120,807 patients from 112 HCOs. The CBD/CBG cohort had a mean age of 66 years (SD = 16), and 55.42% were female. The active-comparator cohort had a mean age of 65 years (SD = 14), and 55.39% were female.

In the pre-chemotherapy exposure analysis, the CBD/CBG cohort included 162 patients from 26 HCOs, while the control cohort included 662,672 patients from 114 HCOs. The pre-chemotherapy CBD/CBG cohort had a mean age of 66 years (SD = 16), and 59.26% were female. The pre-chemotherapy control cohort had a mean age of 67 years (SD = 15), and 54.90% were female. Race and ethnicity distributions are summarized in Table 1.

**Table 1:** Demographic characteristics of post-chemotherapy exposure and pre-chemotherapy exposure cohorts.

Characteristic	Post-chemo CBD/CBG	Post-chemo comparator	Pre-chemo CBD/CBG	Pre-chemo control
Total patients in characteristics export	83	120,807	162	662,672
HCOs	24	112	26	114
Mean age, years ± SD	66 ± 16	65 ± 14	66 ± 16	67 ± 15
Age range	19–90	18–90	18–90	18–90
Female	55.42%	55.39%	59.26%	54.90%
Male	44.58%	44.56%	40.74%	45.05%
Unknown sex	0.00%	0.05%	0.00%	0.05%
Not Hispanic or Latino	62.65%	67.95%	66.04%	57.07%
Hispanic or Latino	12.04%	5.17%	6.17%	4.52%
Unknown ethnicity	34.93%	26.34%	33.33%	38.41%
White	69.87%	61.63%	67.28%	56.12%
Black or African American	12.04%	11.26%	12.34%	8.70%
Asian	12.04%	8.90%	6.17%	9.19%
American Indian or Alaska Native	0.00%	0.44%	6.17%	0.33%
Native Hawaiian or other Pacific Islander	0.00%	0.48%	0.00%	0.54%
Other race	12.04%	4.77%	6.17%	8.19%
Unknown race	16.86%	12.52%	15.43%	16.93%

Descriptive cohort counts reflect TriNetX characteristics exports, whereas Cox model counts reflect patients entering the Cox proportional hazards model after index-event restrictions. Race and ethnicity were extracted from structured electronic health record demographic fields and are reported descriptively to characterize cohort composition. These variables were not interpreted as biological determinants of treatment response.

### Post-chemotherapy CBD/CBG exposure was associated with lower observed hazard of subsequent neuropathy-related diagnosis-code outcomes

Before applying the active-comparator medication exclusion, 173 patients had documented post-chemotherapy CBD/CBG exposure. Of these, 90 patients also had documented gabapentin, pregabalin, or duloxetine exposure in the same post-chemotherapy treatment window and were excluded to create the mutually exclusive CBD/CBG cohort. The post-chemotherapy exposure Cox model included 83 CBD/CBG-exposed patients and 118,001 active-comparator patients. In the adjusted Cox proportional hazards model, CBD/CBG cohort membership was associated with a significantly lower hazard of subsequent neuropathy-related diagnosis compared with the active-comparator cohort: HR = 0.162, 95% CI = 0.041–0.649, *p* = 0.010 (Figure 2). In descriptive outcomes queries, neuropathy-related diagnosis events occurred in 18,290 of 120,067 active-comparator patients. The exact number of neuropathy-related diagnosis events in the post-chemotherapy CBD/CBG cohort could not be displayed because TriNetX suppresses detailed results when outcome counts are between 1 and 9 patients. In a descriptive *post hoc* mortality comparison, death within 365 days after the post-chemotherapy exposure index occurred in 38 of 83 (45.8%) patients in the CBD/CBG cohort and 33,476 of 116,696 (28.7%) patients in the active-comparator cohort, corresponding to a higher observed 365-day mortality proportion in the CBD/CBG cohort than in the active-comparator cohort (risk ratio = 1.596, 95% CI = 1.263–2.017, *p* = 0.0006). Together, these results indicate a lower observed hazard of neuropathy-related diagnosis-code outcomes in the post-chemotherapy CBD/CBG cohort. However, this estimate should be interpreted cautiously because the exposed-arm event count was very small and mortality was more frequent in the CBD/CBG cohort.

**Figure 2:**
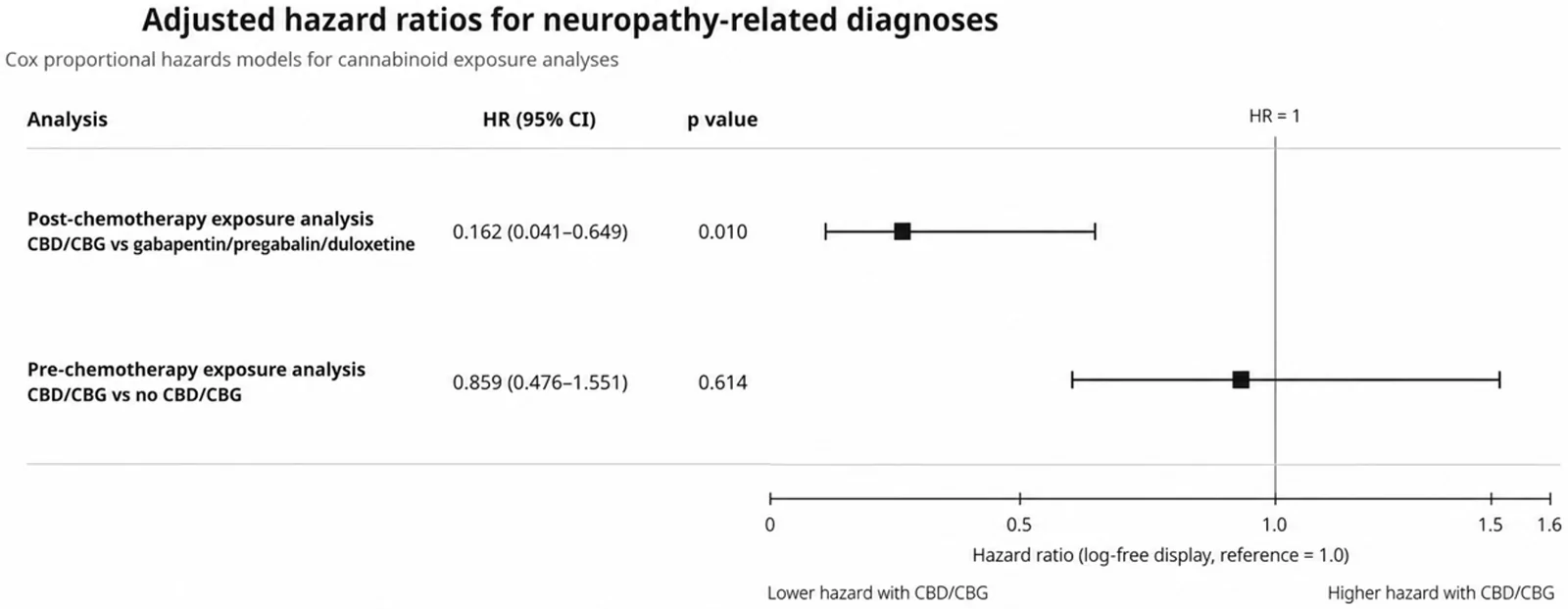
Adjusted hazard ratios for neuropathy-related diagnoses associated with CBD/CBG exposure. The post-chemotherapy exposure analysis compared post-chemotherapy CBD/CBG exposure with active-comparator treatment using gabapentin, pregabalin, or duloxetine. The pre-chemotherapy exposure analysis compared patients with documented CBD/CBG exposure within 1 year before chemotherapy with patients who had no documented CBD/CBG exposure during that pre-chemotherapy window. Hazard ratios less than 1 indicate lower hazard of subsequent neuropathy-related diagnosis in the CBD/CBG cohort. CBD, cannabidiol; CBG, cannabigerol; HR, hazard ratio; CI, confidence interval.

In the post-chemotherapy exposure model, age at index, type 1 diabetes, type 2 diabetes, opioid analgesic exposure, and secondary/unspecified malignant neoplasm of lymph nodes were significantly associated with increased hazard of neuropathy-related diagnosis-code outcomes, whereas male sex and secondary malignant neoplasm of respiratory and digestive organs were significantly associated with lower hazard (Table 2).

**Table 2:** Adjusted Cox proportional hazards model for the post-chemotherapy exposure analysis.

Covariate	Hazard ratio	95% CI	*p* value
CBD/CBG exposure vs. active comparator	0.162	0.041–0.649	0.0101
Male sex	0.837	0.813–0.863	<0.0001
Age at index	1.002	1.001–1.003	<0.0001
Type 1 diabetes mellitus	1.145	1.020–1.285	0.0222
Type 2 diabetes mellitus	1.105	1.065–1.147	<0.0001
Other specified diabetes mellitus	0.965	0.857–1.087	0.5582
Epilepsy and recurrent seizures	0.918	0.806–1.045	0.1937
Convulsions, not elsewhere classified	1.086	0.973–1.213	0.1414
Secondary/unspecified malignant neoplasm of lymph nodes	1.169	1.130–1.209	<0.0001
Secondary malignant neoplasm of respiratory/digestive organs	0.956	0.918–0.995	0.0288
Secondary malignant neoplasm of other/unspecified sites	0.982	0.944–1.021	0.3638
Opioid analgesic exposure	1.274	1.197–1.355	<0.0001

Cox proportional hazards model for neuropathy-related diagnosis-code outcomes occurring 30–365 days after the post-chemotherapy treatment index. The CBD/CBG cohort was compared with an active-comparator cohort defined by post-chemotherapy exposure to gabapentin, pregabalin, or duloxetine. Gabapentin, pregabalin, and duloxetine were not included as covariates in this model because they were exposure-defining comparator medications. Hazard ratios less than 1 indicate lower hazard of neuropathy-related diagnosis-code outcomes.

### Pre-chemotherapy CBD/CBG exposure was not significantly associated with neuropathy-related diagnoses

The pre-chemotherapy Cox model included 162 CBD/CBG-exposed patients and 633,329 control patients after index-event restrictions. In the adjusted Cox model, pre-chemotherapy CBD/CBG exposure was not significantly associated with subsequent neuropathy-related diagnoses (HR = 0.859, 95% CI = 0.476–1.551, *p* = 0.614) (Figure 2). Although the point estimate was below 1, the confidence interval crossed 1, indicating that pre-chemotherapy CBD/CBG exposure was not significantly associated with reduced neuropathy-related diagnoses. In descriptive outcomes queries, neuropathy-related diagnosis events occurred in 12 of 162 pre-chemotherapy CBD/CBG patients and 40,213 of 646,328 control patients.

In the pre-chemotherapy model, type 1 diabetes, type 2 diabetes, metastatic cancer diagnoses, opioid analgesic exposure, gabapentin, pregabalin, and duloxetine were significantly associated with increased hazard of neuropathy-related diagnosis-code outcomes, whereas male sex was significantly associated with lower hazard (Table 3).

**Table 3:** Adjusted Cox proportional hazards model for the pre-chemotherapy exposure analysis.

Covariate	Hazard ratio	95% CI	*p* value
CBD/CBG exposure vs. pre-chemotherapy control	0.859	0.476–1.551	0.6137
Male sex	0.837	0.820–0.854	<0.0001
Age at index	1.000	1.000–1.001	0.3342
Type 1 diabetes mellitus	1.131	1.031–1.240	0.0092
Type 2 diabetes mellitus	1.257	1.223–1.291	<0.0001
Other specified diabetes mellitus	0.977	0.886–1.077	0.6367
Epilepsy and recurrent seizures	0.954	0.863–1.055	0.3627
Convulsions, not elsewhere classified	1.039	0.949–1.138	0.4093
Secondary/unspecified malignant neoplasm of lymph nodes	1.299	1.268–1.331	<0.0001
Secondary malignant neoplasm of respiratory/digestive organs	1.076	1.044–1.108	<0.0001
Secondary malignant neoplasm of other/unspecified sites	1.067	1.036–1.100	<0.0001
Opioid analgesic exposure	1.725	1.678–1.774	<0.0001
Gabapentin	1.631	1.589–1.674	<0.0001
Pregabalin	1.356	1.291–1.424	<0.0001
Duloxetine	1.285	1.214–1.360	<0.0001

Cox proportional hazards model for neuropathy-related diagnosis-code outcomes occurring 30–365 days after the first qualifying neurotoxic chemotherapy exposure in the pre-chemotherapy exposure analysis. The exposed cohort included patients with documented CBD/CBG exposure within 1 year on or before chemotherapy, and the control cohort included patients without documented CBD/CBG exposure during that pre-chemotherapy window. Gabapentin, pregabalin, and duloxetine were included as covariates in this model because pre-index use may reflect baseline pain, neuropathic symptoms, mood disorders, or other comorbidities. Hazard ratios less than 1 indicate lower hazard of neuropathy-related diagnosis-code outcomes.

## Discussion

In this retrospective real-world cohort study, documented post-chemotherapy CBD/CBG exposure was associated with a lower observed hazard of subsequent neuropathy-related diagnosis-code outcomes compared with active-comparator exposure to gabapentin, pregabalin, or duloxetine. However, this association should be interpreted cautiously because the exposed cohort was small, the exposed-arm event count was suppressed because it was between 1 and 9 patients, and mortality within 365 days was more frequent in the CBD/CBG cohort than in the active-comparator cohort. In contrast, pre-chemotherapy CBD/CBG exposure was not significantly associated with neuropathy-related diagnosis-code outcomes. Together, these findings suggest that documented non-THC cannabinoid exposure after neurotoxic chemotherapy may represent a real-world signal for lower subsequent neuropathy-related diagnosis-code outcomes. However, because patients with neuropathy-related diagnoses on or before chemotherapy were excluded, this analysis should not be interpreted as evidence for treatment of established coded CIPN. Conversely, because post-chemotherapy exposure may have occurred in response to emerging symptoms that had not yet been coded, the finding should also not be interpreted as definitive evidence of prevention.

In this model, patients with post-chemotherapy CBD/CBG exposure had a significantly lower hazard of subsequent neuropathy-related diagnoses compared with patients receiving gabapentin, pregabalin, or duloxetine. This active-comparator design is important because it compares CBD/CBG-exposed patients with individuals receiving medications commonly used in clinical practice for neuropathic pain symptoms, rather than comparing against an untreated population. This approach can reduce confounding by indication by making comparator groups more similar with respect to treatment indication and healthcare-seeking behavior (25–27).

The pre-chemotherapy exposure analysis did not identify a statistically significant protective association. Patients with CBD/CBG exposure within one year before chemotherapy showed a directionally lower hazard of neuropathy-related diagnoses, but the confidence interval crossed 1. This finding should not be interpreted as evidence against a prophylactic effect of minor cannabinoids. Rather, the design captures documented exposure before chemotherapy and does not confirm that the medication was continued during chemotherapy, taken at a therapeutic dose, or used specifically for neuropathy prevention. Therefore, the prophylactic analysis is best interpreted as an exploratory pre-chemotherapy exposure analysis rather than a definitive test of continuous cannabinoid prophylaxis.

The difference between the post-chemotherapy exposure and pre-chemotherapy exposure findings may reflect several factors. First, patients receiving CBD/CBG after chemotherapy may represent a group using cannabinoids in closer temporal proximity to the development or treatment of neuropathy-related symptoms, whereas the pre-chemotherapy group may include patients exposed for unrelated indications that were not continued during chemotherapy. Second, structured EHR data do not capture over-the-counter cannabinoid use, product formulation, dose, duration, adherence, or whether the medication contained other cannabinoids. Third, the pre-chemotherapy exposure cohort was small relative to the control cohort, limiting precision. Finally, it is possible that CBD/CBG-related exposure, or related cannabinoid approaches, may be more relevant for established or emerging neuropathy-related symptoms than for pre-chemotherapy prevention, although this possibility requires controlled experimental testing. Preclinical studies suggest that cannabinoids beyond CBD and CBG, including cannabichromene and combined THC/CBD approaches, may also reduce chemotherapy-associated mechanical hypersensitivity or neuropathic pain-like behavior (17, 22, 28, 29). However, these compounds were not evaluated in the present EHR analysis because the structured exposure definition was limited to CBD and CBG concepts.

Current treatment options for CIPN remain limited, and commonly used neuropathic-pain medications provide incomplete or inconsistent relief for many patients (1, 6). In this context, the observed association between post-chemotherapy CBD/CBG exposure and reduced subsequent neuropathy-related diagnoses supports continued investigation of non-THC cannabinoids as candidate therapeutics. Importantly, this study should not be interpreted as evidence that CBD or CBG is clinically effective for CIPN. Rather, it provides a translational signal that complements preclinical work and supports further mechanistic testing of non-THC cannabinoids in controlled models of chemotherapy-induced neuropathy.

The mechanisms by which CBD/CBG-related exposure might influence chemotherapy-associated neuropathy remain incompletely defined, but the biology of CIPN provides several plausible points of convergence. Across neurotoxic chemotherapy classes, CIPN is thought to involve peripheral sensory-neuron injury, mitochondrial dysfunction and oxidative stress, altered ion-channel function, and neuroimmune/inflammatory signaling, which together increase dorsal root ganglion excitability and promote peripheral sensitization (3, 11, 12, 30, 31). These peripheral changes can drive spinal sensitization and altered sensory processing, including recruitment of normally innocuous mechanosensory input into nociceptive circuits, and may propagate to supraspinal pain-processing networks involved in sensory-discriminative and affective dimensions of pain (3, 30–35).

Within this framework, CBD/CBG and related cannabinoid approaches may influence chemotherapy-associated neuropathic symptoms by modulating several levels of the pain pathway rather than through a single receptor mechanism. Mechanistically, these targets map onto several core features of neuropathic pain biology. TLR4 signaling is linked to neuroimmune activation in CIPN (36–39). Endocannabinoid and PPAR*γ* signaling regulate microglial and inflammatory responses in the nervous system, whereas GPR55 influences neurotransmitter release (37, 40–44). In parallel, 5-hydroxytryptamine 1A (5-HT1A) receptor signaling and alpha-2 (*α*2)-adrenergic receptor signaling provide monoaminergic inhibitory mechanisms that can reduce nociceptive transmission, including through presynaptic regulation of neurotransmitter release (45–49). *α*3 glycine receptor potentiation may further strengthen inhibitory control within spinal nociceptive circuits (50–52). Thus, CBD, CBG, and related cannabinoid approaches may reduce neuropathic pain-like behavior by dampening neuroinflammatory signaling, limiting sensory-neuron hyperexcitability, and restoring inhibitory gating of nociceptive input.

For example, CBD has been reported to attenuate paclitaxel-induced mechanical sensitivity through 5-HT1A receptor-dependent mechanisms, and additional work suggests that CBD-related compounds may engage TLR4/endocannabinoid, GPR55, PPAR*γ*-linked, and glycinergic mechanisms relevant to neuropathic pain or chemotherapy neuropathy models (20, 53–57). CBG has been shown to attenuate chemotherapy-induced mechanical hypersensitivity through *α*2-adrenergic mechanisms, and more recent work implicates modulation of thalamocortical pain signaling and neuroinflammatory markers (16, 18, 19, 23, 24). Because CBD, CBG, and related cannabinoids can also intersect with cannabinoid receptor and endocannabinoid signaling, CB2 receptor-targeting approaches provide additional evidence that cannabinoid-sensitive pathways can suppress chemotherapy-associated neuropathic pain-like behavior, although these findings should not be interpreted as evidence for a CBD- or CBG-specific CB2 mechanism (31, 58).

Other cannabinoid approaches beyond CBD/CBG-coded exposure, including combined THC/CBD exposure, THC-enriched vaporized cannabis, CBN, cannabichromene, and cannabinoid-rich Cannabis sativa extracts, have also shown antinociceptive effects in CIPN or broader nociceptive models (17, 22, 28, 29, 32, 59, 60). Notably, the THC-enriched vaporized cannabis study also showed normalization of raphe nucleus hyperconnectivity in a paclitaxel CIPN model, supporting the broader concept that cannabinoid-sensitive interventions may modulate supraspinal pain-processing circuitry (32). However, the present EHR analysis cannot determine whether the observed association reflects a specific cannabinoid, receptor mechanism, product formulation, dose, timing, THC co-exposure, or pathway. Controlled preclinical and prospective clinical studies are therefore needed to determine whether CBD/CBG-related exposure causally modifies chemotherapy-induced neuropathy and to define the relevant mechanisms.

The covariate results also support the face validity of the models. Diabetes was associated with increased hazard of neuropathy-related diagnoses in both analyses, consistent with diabetes being a major documented risk factor for peripheral neuropathy (61). Opioid analgesic exposure was also associated with increased hazard, likely reflecting greater baseline pain burden, more severe symptoms, or increased clinical surveillance (1, 62, 63). In the pre-chemotherapy model, gabapentin, pregabalin, and duloxetine were associated with increased hazard of neuropathy-related diagnoses. These associations should not be interpreted as evidence that these medications cause neuropathy. More likely, they reflect confounding by indication, where patients receiving these medications have baseline pain, early sensory symptoms, or other neurologic complaints that increase the likelihood of subsequent neuropathy coding.

This study has several strengths. It used a large multi–health-care-organization real-world dataset, included multiple neurotoxic chemotherapy classes, excluded patients with neuropathy-related diagnoses before chemotherapy, and evaluated both post-chemotherapy exposure and pre-chemotherapy exposure designs. The active-comparator treatment analysis also reduced some limitations of comparing CBD/CBG-exposed patients with all non-exposed patients.

## Limitations

This observational study cannot establish causality, and residual confounding, confounding by indication, and differences in healthcare utilization may contribute to the observed association. This issue is particularly important for the post-chemotherapy exposure analysis because receipt of gabapentin, pregabalin, or duloxetine after chemotherapy may indicate that patients were already experiencing neuropathic pain, early uncoded CIPN symptoms, or greater baseline pain burden at the time of treatment initiation. Thus, the active-comparator group may have had greater baseline symptom burden or a higher likelihood of neuropathy-related coding than the CBD/CBG group despite covariate adjustment. Excluding patients with both CBD/CBG and gabapentin, pregabalin, or duloxetine exposure created mutually exclusive cohorts but may have selected a distinct CBD/CBG subgroup with different symptom severity, medication preferences, access to cannabinoid products, or clinical documentation patterns.

Additionally, the CBD/CBG-exposed cohorts were small, especially the post-chemotherapy exposure cohort, resulting in wide confidence intervals. Although the Cox models generated finite estimates and confidence intervals, the small CBD/CBG-exposed cohorts may increase vulnerability to sparse-event bias, model instability, and residual confounding. For example, exact event counts for the post-chemotherapy CBD/CBG cohort could not be displayed because TriNetX suppresses detailed results when outcome counts are between 1 and 9 patients. This low exposed-arm event count further underscores the exploratory nature of the estimate and the potential for sparse-event bias and model instability. Therefore, these findings should be interpreted as hypothesis-generating rather than definitive.

Mortality may represent a competing risk for neuropathy-related diagnosis ascertainment in this oncology cohort. Death within 365 days after the post-chemotherapy exposure index was more frequent in the CBD/CBG cohort than in the active-comparator cohort, which may have reduced the opportunity for subsequent neuropathy-related diagnosis coding. Because this analysis did not use a formal competing-risk model, differential mortality between cohorts may have influenced the observed association.

Exposure was limited to structured CBD and CBG medication concepts in the EHR. These records do not reliably capture dose, route, frequency, duration, formulation, indication, adherence, product source, THC content, or whether the documented concept reflected an isolated CBD/CBG product versus a CBD-rich, CBG-containing, or full-spectrum cannabinoid product. Patients with documented THC-containing or other cannabis-related product exposure were not excluded from the primary analyses because structured EHR cannabis-product coding is incomplete and does not reliably distinguish prescription CBD/CBG concepts from over-the-counter, dispensary, CBD-rich, CBG-containing, or full-spectrum products. Excluding only documented THC/cannabis codes would not eliminate unrecorded THC-containing product use and could introduce additional selection bias. Potential co-exposure to THC-containing or full-spectrum cannabinoid products therefore remains an important limitation. Additionally, chemotherapy-agent-specific distributions could not be reliably quantified in the small CBD/CBG-exposed cohorts because chemotherapy exposure was captured using both structured medication and HCPCS procedure concepts, and several agent-specific counts were at or below TriNetX low-count reporting thresholds. Therefore, imbalance in chemotherapy agent, cumulative exposure, dose, cancer type, treatment intent, or concomitant cancer therapy may have influenced the observed associations.

The outcome was based on a diagnosis-code composite rather than clinician-adjudicated CIPN, neuropathy severity scores, patient-reported symptoms, or objective sensory testing. Therefore, the endpoint should be interpreted as a neuropathy-related diagnosis-code outcome rather than confirmed CIPN incidence or symptom severity. The neuropathy-related outcome composite also did not include every ICD-10-CM code potentially related to neuropathy, neuropathic pain, or sensory disturbance. Codes were selected to capture diagnoses plausibly related to chemotherapy-associated neuropathy while avoiding broader pain, neurologic, or disease-specific neuropathy codes that could reduce outcome specificity. As a result, some CIPN-related events may have been missed if they were coded using alternative neuropathy, pain, or sensory-symptom codes, while some captured events may reflect non-CIPN neuropathic or sensory complaints.

Future studies should attempt to replicate this association using alternative datasets, chemotherapy-class-specific analyses, more specific neuropathy outcomes, and designs that better establish cannabinoid exposure timing, persistence, dose, route, formulation, and THC co-exposure. Controlled preclinical studies should directly test whether CBD and CBG reduce chemotherapy-induced mechanical hypersensitivity and alter peripheral or central sensitization, inflammatory signaling, or monoaminergic pain-modulatory pathways. If these findings are replicated, related non-THC cannabinoids could then be prioritized for comparative mechanistic studies.

## Conclusions

Post-chemotherapy CBD/CBG exposure was associated with a significantly lower hazard of subsequent neuropathy-related diagnoses compared with active-comparator neuropathic-pain medications in a real-world EHR cohort. Pre-chemotherapy CBD/CBG exposure was not significantly associated with neuropathy-related outcomes. These findings should be interpreted as hypothesis-generating rather than causal and should not be considered evidence for treatment of established CIPN. Instead, they identify a post-chemotherapy exposure signal that supports continued preclinical and translational investigation of CBD/CBG and related non-THC cannabinoids in chemotherapy-associated neuropathy.

## Data Availability

The raw data supporting the conclusions of this article will be made available by the authors, without undue reservation.
