# Supplementary material for: Cannabidiol/cannabigerol exposure and neuropathy-related outcomes after neurotoxic chemotherapy: a real-world cohort study
Source: Front Pain Res (Lausanne). 2026 Sep 22;7:1945720. doi: 10.3389/fpain.2026.1945720 (PMC13638511; doi:10.3389/fpain.2026.1945720)
Supplement: Supplementary file 1 [file Table1.docx]

**SUPPLEMENTARY TABLES**

**A. Neurotoxic chemotherapy exposure concepts**

| Category | Concept/Drug | Coding system / code | Purpose |
| --- | --- | --- | --- |
| Platinum chemotherapy | Cisplatin | RxNorm 2555 | Exposure anchor |
| Platinum chemotherapy | Cisplatin injection | HCPCS J9060 | Exposure anchor |
| Platinum chemotherapy | Carboplatin | RxNorm 40048 | Exposure anchor |
| Platinum chemotherapy | Carboplatin injection | HCPCS J9045 | Exposure anchor |
| Platinum chemotherapy | Oxaliplatin | RxNorm 32592 | Exposure anchor |
| Platinum chemotherapy | Oxaliplatin injection | HCPCS J9263 | Exposure anchor |
| Taxane chemotherapy | Paclitaxel | RxNorm 56946 | Exposure anchor |
| Taxane chemotherapy | Paclitaxel injection, 1 mg | HCPCS J9267 | Exposure anchor |
| Taxane chemotherapy | Paclitaxel injection, 30 mg | HCPCS J9265 | Exposure anchor |
| Taxane chemotherapy | Paclitaxel protein-bound particles injection, 1 mg | HCPCS J9264 | Exposure anchor |
| Taxane chemotherapy | Paclitaxel protein-bound / nab-paclitaxel, 1 mg | HCPCS J9259 | Exposure anchor |
| Taxane chemotherapy | Paclitaxel protein-bound particles injection, 1 mg | HCPCS J9258 | Exposure anchor |
| Taxane chemotherapy | Docetaxel | RxNorm 72962 | Exposure anchor |
| Taxane chemotherapy | Docetaxel injection | HCPCS J9171 | Exposure anchor |
| Vinca alkaloid | Vincristine | RxNorm 11202 | Exposure anchor |
| Vinca alkaloid | Vincristine injection | HCPCS J9370 | Exposure anchor |
| Proteasome inhibitor | Bortezomib | RxNorm 358258 | Exposure anchor |
| Proteasome inhibitor | Bortezomib injection | HCPCS J9041 | Exposure anchor |
| Proteasome inhibitor | Bortezomib injection (alt formulations) | HCPCS J9046, J9048, J9049, J9051, J9054 | Exposure anchor |

**B. Baseline neuropathy exclusion and outcome concepts**

| ICD-10-CM code | Diagnosis | Used for |
| --- | --- | --- |
| G62.0 | Drug-induced polyneuropathy | Exclusion and outcome |
| G62.9 | Polyneuropathy, unspecified | Exclusion and outcome |
| M79.2 | Neuralgia and neuritis, unspecified | Exclusion and outcome |
| R20.2 | Paresthesia of skin | Exclusion and outcome |
| R20.8 | Other disturbances of skin sensation | Exclusion and outcome |
| R20.9 | Unspecified disturbances of skin sensation | Exclusion and outcome |

**C. Cannabinoid exposure concepts**

| Category | Concept/Drug | Coding system / code | Purpose |
| --- | --- | --- | --- |
| Cannabinoid exposure | Cannabidiol (CBD) | RxNorm 2045371 | Exposure |
| Cannabinoid exposure | Cannabigerol (CBG) | RxNorm 2464938 | Exposure |

**Note**. *Cannabidiol and cannabigerol were the cannabinoid medication concepts selected for analysis because they had sufficient structured capture in TriNetX. These structured concepts should not be interpreted as confirming isolated CBD-only or CBG-only product exposure.*

**D. Active comparator medication concepts**

| Category | Concept/Drug | Coding system / code | Purpose |
| --- | --- | --- | --- |
| Active comparator | Gabapentin | RxNorm 25480 | Comparator / covariate |
| Active comparator | Pregabalin | RxNorm 187832 | Comparator / covariate |
| Active comparator | Duloxetine | RxNorm 72625 | Comparator / covariate |

**E. Covariates**

| Category | Concept | Coding system / code | Purpose |
| --- | --- | --- | --- |
| Demographic covariate | Age at index | TriNetX demographic variable | Adjustment |
| Demographic covariate | Sex | TriNetX demographic variable | Adjustment |
| Diabetes | Type 1 diabetes mellitus | ICD-10-CM E10.* | Adjustment |
| Diabetes | Type 2 diabetes mellitus | ICD-10-CM E11.* | Adjustment |
| Diabetes | Other specified diabetes mellitus | ICD-10-CM E13.* | Adjustment |
| Neurologic covariate | Epilepsy and recurrent seizures | ICD-10-CM G40.* | Adjustment |
| Neurologic covariate | Convulsions | ICD-10-CM R56.* | Adjustment |
| Pain covariate | Opioid analgesic exposure | VA CN101 | Adjustment |
| Metastatic disease | Secondary and unspecified malignant neoplasm of lymph nodes | ICD-10-CM C77.* | Adjustment |
| Metastatic disease | Secondary malignant neoplasm of respiratory and digestive organs | ICD-10-CM C78.* | Adjustment |
| Metastatic disease | Secondary malignant neoplasm of other and unspecified sites | ICD-10-CM C79.* | Adjustment |
| Baseline pain medication covariates* | Gabapentin, pregabalin, duloxetine | RxNorm: 25480, 187832, 72625 | Pre-chemotherapy model only |

**Note.** Gabapentin, pregabalin, and duloxetine were included as covariates in the pre-chemotherapy exposure analysis, but not in the final post-chemotherapy exposure active-comparator model because they were exposure-defining comparator medications. An asterisk after an ICD-10-CM category indicates inclusion of all descendant codes within that category.

**Supplementary Tables Note.** Concepts were identified in the TriNetX platform using structured diagnosis, medication, and procedure terminology. Medication concepts included RxNorm and/or platform-specific structured medication concepts, and chemotherapy administration concepts included HCPCS procedure codes where applicable.
